# Impact of Short-Term Continuous and Interval Exercise Training on Endothelial Function and Glucose Metabolism in Prediabetes

**DOI:** 10.1155/2019/4912174

**Published:** 2019-12-28

**Authors:** Steven K. Malin, Nicole M. Gilbertson, Natalie Z. M. Eichner, Emily Heiston, Stephanie Miller, Arthur Weltman

**Affiliations:** ^1^Department of Kinesiology, University of Virginia, Charlottesville, VA, USA; ^2^Division of Endocrinology & Metabolism, Department of Medicine, University of Virginia, Charlottesville, VA, USA; ^3^Robert M. Berne Cardiovascular Research Center, University of Virginia, Charlottesville, VA, USA

## Abstract

**Introduction:**

The impact of interval (INT) vs. continuous (CONT) exercise training on endothelial function in relation to glucose metabolism prior to clinically meaningful weight loss is unknown in adults with prediabetes.

**Methods:**

Twenty-six subjects with prediabetes (60 ± 1 y; 33 ± 1 kg/m^2^; 2-hr-PG OGTT: 145 ± 7 mg/dl) were randomized to 60 min of CONT (*n* = 12; 70% of HR_peak_) or work-matched INT exercise training (*n* = 14; alternating 3 min at 90 and 50% HR_peak_) for 2 weeks. Aerobic fitness (VO_2peak_) and body composition (bioelectrical impedance) were assessed before and after training. Flow-mediated dilation (FMD) was measured during a 2 h 75 g OGTT (0, 60, and 120 min) to assess endothelial function. Postprandial FMD was calculated as incremental area under the curve (iAUC). Glucose tolerance and insulin were also calculated by iAUC. Fasting plasma VCAM, ICAM, and hs-CRP were also assessed as indicators of vascular/systemic inflammation.

**Results:**

Both interventions increased VO_2peak_ (*P* = 0.002) but had no effect on body fat (*P* = 0.20). Although both treatments improved glucose tolerance (*P* = 0.06) and insulin iAUC (*P* = 0.02), VCAM increased (*P* = 0.01). There was no effect of either treatment on ICAM, hs-CRP, or fasting as well as postprandial FMD. However, 57% of people improved fasting and iAUC FMD following CONT compared with only 42% after INT exercise (each: *P* = 0.04). Elevated VCAM was linked to blunted fasting FMD after training (*r* = −0.38, *P* = 0.05). But, there was no correlation between fasting FMD or postprandial FMD with glucose tolerance (*r* = 0.17, *P* = 0.39 and *r* = 0.02, *P* = 0.90, respectively) or insulin iAUC following training (*r* = 0.34, *P* = 0.08 and *r* = 0.04, *P* = 0.83, respectively).

**Conclusion:**

Endothelial function is not improved consistently after short-term training, despite improvements in glucose and insulin responses to the OGTT in obese adults with prediabetes.

## 1. Introduction

Impaired endothelial function is a leading candidate for macro- and microvascular disturbances that contribute, in part, to the 50% higher risk of cardiovascular disease (CVD) in adults with prediabetes when compared with healthy controls [1]. Attenuated endothelial function in particular can occur during the postprandial state as a result of hyperglycemia-induced inflammation that decreases nitric oxide and elevates endothelin-1 [2, 3]. This later point is clinically meaningful since postprandial hyperglycemia is a stronger predictor of future CVD and mortality than fasting glucose [4, 5]. Therefore, restoring fasting and postprandial vascular function may be important for improving glucose regulation and lowering chronic disease risk.

Exercise training is established to raise insulin-mediated skeletal muscle glucose uptake [6, 7] and limb blood flow, thereby contributing to reduced cardiometabolic disease risk [8]. We recently reported that 2 weeks of interval (INT) exercise when compared with continuous (CONT) training induced similar improvement in glucose tolerance [9] as well as arterial stiffness in people with prediabetes [10]. However, neither insulin sensitivity nor arterial stiffness related to this improved glucose tolerance. Interestingly, our group previously demonstrated that a single bout of high-intensity exercise, when compared with moderate intensity exercise, increased glucose tolerance in only individuals with low endothelial function as measured by flow-mediated dilation (FMD) [11]. This reinforces the view that not all people respond the same way to a given exercise bout [12, 13] and exercise may be a key for vascular function-mediated glycemic control in people with prediabetes. Although recent work suggests that INT exercise improves FMD more than CONT exercise in sedentary people [14–16], not all studies agree [17]. This later finding in fact suggested CONT exercise increased FMD more than INT, which is consistent with several reports showing that either a single bout or short-term exercise training at moderate CONT intensity can restore/improve vasodilation following glucose ingestion in people with and without type 2 diabetes [18–21]. To date, however, no study has tested whether CONT exercise increases FMD before or after an OGTT to a similar extent as INT training when matched on energy expenditure and before clinically relevant weight loss. Thus, we tested the hypothesis that CONT exercise would increase endothelial function before and after glucose ingestion as much as INT training in obese adults with prediabetes. We also sought to determine if changes in FMD related to improved glucose metabolism.

## 2. Methods

### 2.1. Subjects

Subjects were recruited via social media and/or newspaper flyers from the local community. Some subjects with prediabetes (*n* = 26; 60 ± 1 y; 33 ± 1 kg/m^2^; 2-hr-PG OGTT: 145 ± 7 mg/dl) were the same individuals that were previously reported in our randomized study on glucose tolerance [9]. Prediabetes was defined according to American Diabetes Association guidelines as either a fasting plasma glucose of 100-126 mg/dl and/or 2-hr glucose between 140 and 199 mg/dl after a 75 g oral glucose tolerance test (OGTT) since no work has shown FMD difference between these phenotypes. Subjects were excluded if they were smoking, physically active (exercise > 60 min/wk), and weight unstable (>2 kg last 3 months), had chronic disease (e.g., type 2 diabetes, cardio-pulmonary dysfunction, etc.), or are taking medications considered to impact endothelial function or insulin sensitivity (e.g., beta-blockers, ACE-inhibitors, metformin, and SGLT-2 inhibitors) based on phone screening with questionnaires. Blood and urine analyses as well as medical history and physical exam were conducted. All subjects provided written and verbal informed consent as approved by our Institutional Review Board.

### 2.2. Body Composition and Aerobic Fitness

Body mass was measured on a digital scale and height was recorded with a stadiometer to determine body mass index (BMI). Body fat and fat-free mass were measured by bioelectrical impedance (InBody 770 Analyzer, Cerritos, CA) in our Applied Metabolism & Physiology Laboratory [22]. Subjects completed a continuous incremental peak oxygen consumption (VO_2peak_) and heart rate (HR_peak_) test using standard criteria on cycle ergometer with indirect calorimetry (Carefusion, Vmax Encore, Yorba Linda, CA) in the Exercise Physiology Core Laboratory as previously described [9].

### 2.3. Metabolic Control

Subjects were instructed to consume a mixed diet containing about 250 g of carbohydrates during the 24 hr period prior to the preintervention testing. This dietary pattern was recorded and replicated on the day before posttesting. Three-day food logs, including two weekdays and one weekend day, were used to assess ad libitum food intake before and after training (ESHA Research, Version 11.1, Salem, OR). Subjects were also instructed to refrain from alcohol, caffeine, medication, and strenuous physical activity for 24 hr prior to each study visit.

### 2.4. Endothelial Function and Metabolism

After an overnight fast, subjects reported to the Clinical Research Unit. Subjects rested in a semisupine position while an intravenous line was placed in the antecubital vein for blood collection. Blood samples were obtained for the determination of plasma glucose as well as insulin at 0, 30, 60, 90, and 120 minutes of the 75 g OGTT. Incremental area under the curve (iAUC) was calculated using the trapezoidal model to determine glucose tolerance and ambient insulin concentrations. Fasting plasma was collected to measure high-sensitivity C-reactive protein (hs-CRP), vascular cell adhesion molecule 1 (VCAM-1), and intercellular adhesion molecule 1 (ICAM-1) to assess systemic and vascular inflammation. Endothelial function was assessed via FMD at the brachial artery using an Epiq 7C Ultrasound Machine (Philips Medical Systems, Andover, MA). Measurements were collected when participants were fasted as well as at 60 and 120 minutes during the OGTT. FMD was defined as the percent change in peak diameter compared to the baseline diameter. FMD iAUC was also calculated to depict postprandial endothelial function. Systolic and diastolic brachial artery blood pressure was also measured at 0, 60, and 120 minutes of the OGTT using the SphygmoCor® XCEL system (AtCor Medical, Itasca, IL) while subjects were semisupine.

### 2.5. Endothelial Function Analysis

All brachial artery assessments were performed in a quiet room, with participants in the supine position, and on the left arm. The brachial artery was imaged with a 12-3 MHz range linear transducer approximately 5 cm proximal to the antecubital crease using B-mode ultrasound. The same investigator completed all measures during the OGTT before and after training. A blood pressure cuff was placed around the forearm distal to the olecranon process. Following baseline brachial artery diameter imaging, the blood pressure cuff was inflated manually to 200 mmHG for 5 minutes and then deflated. The diameter of the brachial artery was imaged every 5 seconds postdeflation for 2 minutes to capture postischemic peak diameter. All images were stored in Digital Imagining and Communication in Medicine (DICOM) format for analyses. Brachial artery images were analyzed by a single investigator (S. L. Miller) blinded to the conditions using commercially available software (Brachial Analyzer for Research v.6, Medical Imaging Applications LLC, Coralville, IA). The software uses an automated method for near and far wall border detection and vessel diameter measurement in brachial ultrasound image sequences. Arterial diameter was measured as the distance between the intima-lumen interfaces of the anterior and posterior walls.

### 2.6. Exercise Training

Subjects were randomly assigned to 12, 60 minute/d work-matched bouts of CONT or INT cycle ergometry exercise over 13 days as previously described [10]. In short, CONT exercise was performed at a constant intensity of 70% HR_peak_, whereas INT exercise involved alternating 3 minute intervals at 90% HR_peak_ followed by 50% HR_peak_. HR (Polar Electro, Inc. Woodbury, NY), and rating of perceived exertion (RPE) was also monitored throughout training. Exercise energy expenditure was calculated using HR-VO2 regression analysis [10]. For CONT exercise, the equation *Y* = 0.0165*X* − 0.7391 and *Y* = 0.0141*X* − 0.4372 were used, respectively, for days 0-7 and 8-13 of training to account for fitness adaptations. A similar approach was taken for INT training: *Y* = 0.0237*X* − 1.4149 and *Y* = 0.203*X* − 1.1329. Posttest assessments were obtained approximately 24 hr after the last training session.

### 2.7. Biochemical Analysis

Plasma glucose was analyzed by a glucose oxidase assay (YSI Instruments 2700, Yellow Springs, OH). Remaining samples were centrifuged at 4°C for 10 min at 3000 RPM and stored at -80°C until analysis. Plasma insulin (Millipore, Billerica, MA) as well as hs-CRP, VCAM, and ICAM (R&D Systems, Minneapolis, MN) were batched analyzed in duplicate to minimize variance within conditions using ELISA.

### 2.8. Statistical Analysis

Data were analyzed using the statistical program R (Vienna, Austria 2013). Based on prior INT exercise (delta 3.85%, SD 2.8%) [16] work on fasting FMD in obese adults, it was determined that 9 participants (80% power and alpha of 0.05) would be needed to determine statistical significance between CONT and INT. Data not meeting normality were log transformed for statistical analysis. Baseline data were compared with independent *t*-tests. Data were compared across conditions using repeated measures analysis of variance (ANOVA) and two-way repeated measures ANOVA (group × test). Cohen's *d* effect sizes were also calculated on the interaction of treatments, and physiological relevance was interpreted as small (*d* = 0.2), medium (*d* = 0.5), or large (*d* = 0.8). Bivariate regression analysis was used to determine associations. Due to intersubject variation in response to exercise, a preliminary analysis of individuals considered responders were considered FMD changes above 0 as we, and others, previously performed [12, 23]. Fischer's exact test was then used to identify statistical significance of responder and nonresponders for group analysis. Statistical significance was accepted as *P* ≤ 0.05. Data are reported as mean ± SEM.

## 3. Results

### 3.1. Subject Characteristics

Although individuals in the CONT training group were heavier at preintervention when compared with those undergoing INT exercise (*P* < 0.05; Table 1), both CONT and INT exercise reduced body weight comparably by approximately 0.5 kg (*P* = 0.001). However, there was no difference following CONT or INT training on body fat (Table 1). VO_2peak_ increased following both CONT and INT (*P* = 0.002, Table 1). There were 8 women in both CONT and INT that were postmenopausal, and 3 women in CONT that were perimenopausal. Exercise adherence to CONT and INT training were excellent (96.1 ± 2.2 vs. 95.9 ± 1.6%, *P* = 0.93). Although the percent of HR_peak_ was approximately 73 ± 1% and 77 ± 1% for CONT and INT, respectively (*P* = 0.01), subjects had similar RPE (12.9 ± 0.3 vs. 11.8 ± 0.5 a.u.; *P* = 0.12) and exercise energy expenditure (384.4 ± 18.2 vs. 396.1 ± 19.5 kcal/session, *P* = 0.66). There were no ad libitum caloric, fiber, fat, or protein intake differences posttraining (data not shown), although carbohydrate intake was lower after INT compared with CONT exercise (−43.2 ± 19.6 vs. 17.1 ± 15.2 g, *P* = 0.02, effect size: 0.89) and this was driven by reductions in dietary sugar (−45.2 ± 11.6 vs. 16.6 ± 8.6 g, *P* < 0.001, effect size: 1.3).

### 3.2. Glucose Metabolism and Inflammation

CONT and INT training had no effect on fasting glucose (*P* = 0.48), but reduced 2-hr glucose concentrations (*P* = 0.02) and tended to lower glucose iAUC (*P* = 0.06; Table 1). Although CONT and INT exercise had no effect on fasting insulin, training tended to reduce 2-hr insulin (*P* = 0.08) and insulin iAUC (*P* = 0.02; Table 1). In addition, VCAM, but not ICAM or hs-CRP, was significantly increased after CONT and INT exercise (*P* = 0.01, Table 1).

### 3.3. Endothelial Function and Blood Pressure

**Figure 1:**
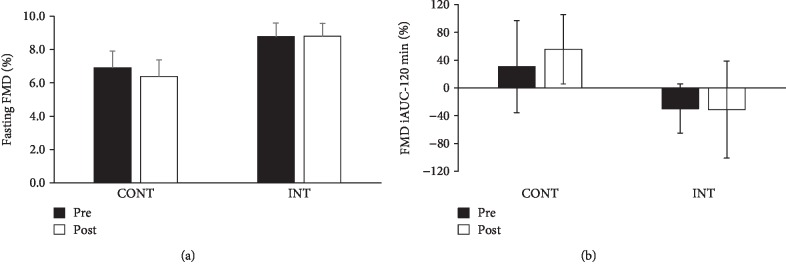
Effect of exercise intensity on endothelial function. Data are mean ± SEM. (a) Fasting flow-mediated dilation (FMD). (b) Postprandial incremental area under the curve (iAUC) for FMD. Effect size for fasting and iAUC FMD was 0.04 and 0.11, respectively.

There was no difference in baseline brachial artery diameter between CONT and INT training. However, arterial diameter was lower following both treatments at 60 minutes of the OGTT (Table 2). Fasting and postprandial FMD were also not statistically different after CONT and INT exercise (Figures 1(a) and 1(b)). However, there was large intersubject variation in response to both interventions. Interestingly, 57% of people undergoing CONT exercise responded by increasing fasting and iAUC FMD compared with 42% after INT (each *P* = 0.04). There was no effect of exercise training on fasting or postprandial systolic or diastolic blood pressure (Table 2).

### 3.4. Correlation Analysis

Increased circulating VCAM was linked to reduced fasting FMD (*r* = −0.38, *P* = 0.05). There was no correlation between fasting FMD or postprandial FMD with glucose tolerance after the intervention (*r* = 0.17, *P* = 0.39 and *r* = 0.02, *P* = 0.90, respectively). In addition, the change in insulin iAUC following training did not correlate with changes in fasting FMD (*r* = 0.34, *P* = 0.08) or FMD iAUC (*r* = 0.04, *P* = 0.83). Enhanced VO_2peak_ also did not correlate with rises in fasting FMD (*r* = −0.14, *P* = 0.46) or FMD iAUC (*r* = −0.26, *P* = 0.19). Changes in fasting FMD or FMD iAUC did not correlate with reductions in dietary total carbohydrate (*r* = 0.10, *P* = 0.60 and *r* = −0.16, *P* = 0.28) or sugar (*r* = 0.20, *P* = 0.33 and *r* = −0.22, *P* = 0.28). Decreased FFM also did not correlate with rises in fasting FMD (*r* = −0.07, *P* = 0.73) or FMD iAUC (*r* = −0.18, *P* = 0.37). Low preintervention fasting FMD correlated with increased posttraining fasting FMD (*r* = −0.81, *P* < 0.01). Preintervention FMD iAUC correlated with the change in posttraining FMD iAUC (*r* = −0.55, *P* < 0.01).

## 4. Discussion

The primary finding in this study was that CONT and INT exercise for 2 weeks did not increase endothelial function in obese people with prediabetes despite improvements in glucose tolerance and insulin responses to the OGTT. These findings suggest that 2 weeks of exercise training may not be sufficient to improve FMD in obese adults with prediabetes. Interestingly, single bouts or training for as short as 7 days of CONT moderate intensity exercise has been reported to improve vascular function during fasting and periods of hyperglycemia [17–21]. It could be speculated that higher intensities of exercise may be needed in older, sedentary obese adults with prediabetes to elicit a rise in FMD given literature suggesting that INT is the “superior” form of exercise [15]. However, INT exercise did not consistently raise FMD in our study either. This observation should not be completely surprising though since high-intensity exercise has been reported to impair [24] or have no effect [25] on fasting endothelial function. It is beyond the scope of this study to determine exactly why FMD did not increase after training, but our study observed no differences in baseline diameter or blood pressure across the OGTT following the intervention. These later findings suggest it is unlikely that arterial remodeling/size [26] or force of blood flow played a role. Another possible reason may relate to exercise-induced inflammation impairing vasodilation via blunted nitric oxide bioavailability [25, 27, 28]. Herein, CONT and INT exercise increased plasma VCAM, and the elevations in VCAM after training correlated with attenuated rises in fasting FMD. These data agree with recent work in people with type 2 diabetes showing that 6 weeks of resistance exercise raised circulating TNF-*α* and VCAM in parallel with no improvement in FMD [29]. Further work is warranted to understand the physiologic importance of this vascular inflammation as it relates training adaptation and cardiometabolic health.

A clinically relevant observation of this study was that short-term exercise training improved postprandial glucose tolerance in obese adults with prediabetes. Postprandial glucose is regulated, in part, by distribution of blood flow, hepatic glucose production, and peripheral glucose uptake [30]. Since hyperglycemia is established to impair endothelial function [20, 21, 31], it would be reasonable to expect that better glucose tolerance would correlate with elevations in FMD. However, we identified no such association between FMD and ambient glucose levels. Although this suggests that changes in large conduit arteries do not relate to improved glucose tolerance after short-term training, we cannot rule out the possibility that vascular function influenced plasma glucose since changes in nutrient exchange mostly reflect the microcirculation [30, 32]. Recent work showed that resistance exercise training for 6 weeks increased skeletal muscle microcirculatory function during an OGTT independent of FMD in adults with type 2 diabetes [33]. Notwithstanding this, the improved glucose tolerance in our study after training could be due to improved insulin secretion and action on hepatic glucose production or peripheral glucose uptake [34]. Since fasting glucose is known to be primarily determined by hepatic glucose production, and we observed no change in fasting glucose (or insulin), our results support that peripheral adaptations likely explain the lowering of plasma glucose after training [32]. Supporting this hypothesis is the observation that postprandial hyperinsulinemia was reduced after both CONT and INT exercise training, suggesting improved peripheral insulin sensitivity. This is consistent with previous work from our group showing that independent of intensity, training increased carbohydrate use during an OGTT in relation to improved glucose tolerance [9].

The limitations of this study warrant discussion. We acknowledge that measures of FMD were performed in the brachial artery and exercise was performed on the cycle ergometer. Electing this mode of exercise may have caused lower limb regional or tissue-specific adaptations that were not captured by assessing upper extremity endothelial function [35]. However, lower limb training has been reported to raise upper limb endothelial function [36]. We also recognize that FMD was not corrected for shear stress or allometric scaling in the current study as performed by some [37–40] but not all [41]. Although these scaling methods may help isolate flow-mediated response understandings [42], the relationship between shear stress and age has been questioned [40]. Even correcting for baseline arterial differences between individuals has proposed biologic concerns, suggesting that there is not currently a clear standard method for assessing FMD at this time [43, 44]. For instance, increased arterial distensibility has been suggested to decrease shear stress by nearly 30% compared with more rigid arteries [45]. Given that we noted differences in arterial stiffness [10] following 2 weeks of CONT and INT exercise in a similar cohort of adults, it is possible that arterial compliance impacted a key stimulus for FMD. Nevertheless, FMD uncorrected for shear stress is an independent predictor of CVD risk and mortality [46], and we studied a relatively homogenous population of older, obese adults before and after treatment under similar conditions (e.g., time of day and rest period). Additional work though is needed using more sophisticated approaches that examine FMD after exercise [39]. Previous research highlighted that not all people increase FMD after exercise [12]. As a preliminary analysis to understand why we did not detect significant changes in FMD after training, we noted that more people increased fasting and postprandial FMD in response to CONT compared with INT training. The term responders here should be interpreted with caution, as the classification is likely to be outcome specific. For example, people who are considered to be nonresponders in the current study based on FMD likely experienced other multiple benefits of exercise (i.e., VO_2peak_ and glycemia). Additionally, like prior work [12], we show that individuals with low endothelial function during fasting or postprandial conditions are likely to improve after training. While our study is of modest sample size, our results suggest that precision exercise medicine investigation is warranted. The subtle weight loss in this study was attributable to declines in FFM since fat mass was unchanged. It should be noted though that multifrequency bioelectrical impendence was used to assess body composition, and this approach is susceptible to water loss. Regardless, the slight declines in FFM were not statistically related to FMD, suggesting that FMD responses were not due to changes in FFM. Prior groups [47] have reported that postmenopausal women do not respond favorably to exercise due to loss of estrogen. In turn, the lack of overall training response seen after INT or CONT exercise may be the result of most women in the study being postmenopausal. Moreover, menstrual cycle was not strictly controlled for in the current study given the 2-week timeframe and recent work suggesting no FMD menstrual phase effect [48].

In conclusion, endothelial function was not improved following 2 weeks of CONT or INT exercise in people with prediabetes despite improved glucose tolerance and circulating insulin concentrations. Interestingly, higher vascular inflammation after training as reflected by VCAM did correlate with decreased fasting vascular function. Thus, further work is warranted to understand how exercise impacts vascular function across the arterial tree to gain better understanding of how exercise delays, prevents, and/or treats type 2 diabetes and CVD.

## Figures and Tables

**Table 1:** Effect of a 2-week INT and CONT exercise training on subject characteristics.

	CONT		INT		ANOVA (*P* value)		Effect size
	Pre	Post	Pre	Post	Test	*G* × *T*	Cohen's *d*
Subjects (F/M)	14 (11/3)	—	12 (9/3)	—	—	—	—
Age (years)	60.4 ± 2.3	—	59.9 ± 2.2	—	—	—	—
Post-menopausal (*n*)	8	—	8	—	—	—	—
Body composition							
Weight (kg)	97.7 ± 3.6^	97.3 ± 3.5	85.6 ± 3.4	84.5 ± 3.4	0.001	0.12	0.61
BMI (kg m^−2^)	35.6 ± 1.6^	34.4 ± 1.6	30.9 ± 1.1	30.5 ± 1.1	0.002	0.33	0.38
Fat mass (kg)	44.0 ± 3.0^	44.2 ± 3.0	34.6 ± 2.4	34.4 ± 2.6	0.83	0.31	0.06
FFM (kg)	53.7 ± 2.1	53.0 ± 2.1	53.2 ± 3.2	52.3 ± 3.2	<0.001	0.40	0.33
Aerobic fitness							
VO_2peak_ (ml kg^−1^ min^−1^)	19.9 ± 1.6	19.8 ± 1.7	20.7 ± 0.8	22.9 ± 0.9	0.002	0.07	0.70
Metabolism							
Glucose (mg dl^−1^)							
Fasting	98.7 ± 4.0	98.4 ± 4.1	101.3 ± 1.8	102.5 ± 2.4	0.48	0.18	0.53
120 min	141.9 ± 8.2	130.1 ± 7.3	142.4 ± 11.7	131.9 ± 10.5	0.02	0.67	0.17
120 min iAUC	6789.6 ± 924.5	5546.0 ± 873.1	5186.3 ± 836.9	4704.0 ± 572.6	0.07	0.42	0.32
Insulin (*μ*U ml^−1^)							
Fasting	13.3 ± 2.0	14.2 ± 2.3	12.5 ± 2.5	12.0 ± 2.3	0.74	0.40	0.34
120 min	105.8 ± 15.5	90.3 ± 11.8	88.1 ± 19.4	75.4 ± 16.0	0.08	0.86	0.07
120 min iAUC	8947.5 ± 1192.9	7458.1 ± 1122.9	8767.3 ± 1123.3	7888.1 ± 1145.7	0.01	0.50	0.27
Inflammation							
VCAM (n ml^−1^)^∗^	622.8 ± 38.1	638.2 ± 45.9	610.6 ± 42.1	652.8 ± 41.9	0.01	0.97	0.09
ICAM (ng ml^−1^)	200.6 ± 12.9	188.9 ± 14.0	217.4 ± 19.0	207.4 ± 19.0	0.48	0.39	0.36
hs-CRP (ng ml^−1^)^∗^	4.8 ± 1.4	4.2 ± 1.0	2.8 ± 0.5	3.2 ± 0.8	0.74	0.50	0.26

Data are mean ± SEM. BMI: body mass index; FFM, fat-free mass; VO2_peak_: peak oxygen consumption; iAUC: incremental area under the curve; hs-CRP: high-sensitivity C-reactive protein; VCAM-1: vascular cell adhesion molecule 1; ICAM-1: intercellular adhesion molecule 1. ^∗^Raw values are presented but data were log transformed for statistical analysis. ^^^Compared with INT pretest, *P* < 0.05.

**Table 2:** Effect of CONT and INT exercise training on preocclusion brachial artery diameter and blood pressure.

	CONT		INT		ANOVA (*P* value)		Effect size
	Pre	Post	Pre	Post	*T*	*G* × *T*	Cohen's *d*
0 min diameter (mm)	3.80 ± 0.18	3.70 ± 0.15	3.84 ± 0.23	3.77 ± 0.18	0.23	0.81	0.06
60 min diameter (mm)	3.87 ± 0.14	3.74 ± 0.14	3.97 ± 0.17	3.92 ± 0.18	0.02	0.33	0.40
120 min diameter (mm)	3.93 ± 0.16	3.95 ± 0.14	3.86 ± 0.16	3.88 ± 0.17	0.71	0.92	0.04
0 min SBP (mmHg)	146.9 ± 4.6	139.0 ± 4.25	133.5 ± 3.7	133.5 ± 4.0	0.20	0.17	0.55
60 min SBP (mmHg)	144.3 ± 4.6	143.0 ± 4.4	137.7 ± 4.7	131.4 ± 4.0	0.25	0.47	0.29
120 min SBP (mmHg)	144.2 ± 5.9	141.3 ± 4.1	133.8 ± 4.2	133.6 ± 4.6	0.63	0.77	0.07
0 min DBP (mmHg)	85.0 ± 5.9	81.0 ± 2.3	79.2 ± 2.8	79.8 ± 3.0	0.46	0.26	0.45
60 min DBP (mmHg)	80.0 ± 2.6	78.3 ± 2.7	75.2 ± 2.8	73.1 ± 2.2	0.28	0.98	0.03
120 min DBP (mmHg)	80.1 ± 3.5	79.7 ± 3.1	77.5 ± 2.0	76.3 ± 2.7	0.63	0.85	0.09

Data are mean ± SEM. SBP: systolic blood pressure; DBP: diastolic blood pressure.

## Data Availability

Data used to support this study may be requested upon reasonable request from the corresponding author.

## References

[1] DeFronzo R., Abdul G. M. (2011). Assessment and treatment of cardiovascular risk in prediabetes: impaired glucose tolerance and impaired fasting glucose. *The American Journal of Cardiology*.

[2] Baron A. D., Laakso M., Brechtel G., Hoit B., Watt C., Edelman S. V. (1990). Reduced postprandial skeletal muscle blood flow contributes to glucose intolerance in human obesity. *The Journal of Clinical Endocrinology & Metabolism*.

[3] Clark M. G. (2008). Impaired microvascular perfusion: a consequence of vascular dysfunction and a potential cause of insulin resistance in muscle. *American Journal of Physiology-Endocrinology and Metabolism*.

[4] Anonymous Glucose tolerance and mortality: comparison of WHO and American Diabetes Association diagnostic criteria (1999). The DECODE study group. European Diabetes Epidemiology Group. Diabetes Epidemiology: Collaborative analysis of Diagnostic criteria in Europe. *The Lancet*.

[5] Cavalot F., Petrelli A., Traversa M. (2006). Postprandial blood glucose is a stronger predictor of cardiovascular events than fasting blood glucose in type 2 diabetes mellitus, particularly in women: lessons from the San Luigi Gonzaga Diabetes Study. *The Journal of Clinical Endocrinology & Metabolism*.

[6] Malin S. K., Niemi N., Solomon T. P. J. (2012). Exercise Training with Weight Loss and either a High- or Low-Glycemic Index Diet Reduces Metabolic Syndrome Severity in Older Adults. *Annals of Nutrition and Metabolism*.

[7] Utriainen T., Mäkimattila S., Virkamäki A., Lindholm H., Sovijärvi A., Yki-Järvinen H. (1996). Physical fitness and endothelial function (nitric oxide synthesis) are independent determinants of insulin-stimulated blood flow in normal subjects. *The Journal of Clinical Endocrinology & Metabolism*.

[8] Ebeling P., Bourey R., Koranyi L. (1993). Mechanism of enhanced insulin sensitivity in athletes. Increased blood flow, muscle glucose transport protein (GLUT-4) concentration, and glycogen synthase activity. *Journal of Clinical Investigation*.

[9] Gilbertson N. M., Eichner N. Z. M., Francois M. (2018). Glucose tolerance is linked to postprandial fuel use independent of exercise dose. *Medicine & Science in Sports & Exercise*.

[10] Eichner N. Z. M., Gaitan J. M., Gilbertson N. M., Khurshid M., Weltman A., Malin S. K. (2019). Postprandial augmentation index is reduced in adults with prediabetes following continuous and interval exercise training. *Experimental Physiology*.

[11] Malin S. K., Rynders C. A., Weltman J. Y., Jackson Roberts L., Barrett E. J., Weltman A. (2016). Endothelial function following glucose ingestion in adults with prediabetes: role of exercise intensity. *Obesity*.

[12] Green D., Eijsvogels T., Bouts Y. (2014). Exercise training and artery function in humans: nonresponse and its relationship to cardiovascular risk factors. *Journal of Applied Physiology*.

[13] Swift D., Weltman J., Patrie J. (2014). Predictors of improvement in endothelial function after exercise training in a diverse sample of postmenopausal women. *Journal of Women's Health*.

[14] Ramirez-Velez R., Hernandez-Quinones P. A., Tordecilla-Sanders A. (2019). Effectiveness of HIIT compared to moderate continuous training in improving vascular parameters in inactive adults. *Lipids in Health and Disease*.

[15] Ramos J. S., Dalleck L. C., Tjonna A. E., Beetham K. S., Coombes J. S. (2015). The impact of high-intensity interval training versus moderate-intensity continuous training on vascular function: a systematic review and meta-analysis. *Sports Medicine*.

[16] Sawyer B. J., Tucker W. J., Bhammar D. M., Ryder J. R., Sweazea K. L., Gaesser G. A. (2016). Effects of high-intensity interval training and moderate-intensity continuous training on endothelial function and cardiometabolic risk markers in obese adults. *Journal of Applied Physiology*.

[17] Shenouda N., Gillen J. B., Gibala M. J., Mac Donald M. J. (2017). Changes in brachial artery endothelial function and resting diameter with moderate-intensity continuous but not sprint interval training in sedentary men. *Journal of Applied Physiology*.

[18] Fujita S., Rasmussen B. B., Cadenas J. G. (2007). Aerobic exercise overcomes the age-related insulin resistance of muscle protein metabolism by improving endothelial function and Akt/mammalian target of rapamycin signaling. *Diabetes*.

[19] Mikus C., Fairfax S., Libla J. (2011). Seven days of aerobic exercise training improves conduit artery blood flow following glucose ingestion in patients with type 2 diabetes. *Journal of Applied Physiology*.

[20] Weiss E., Arif H., Villareal D., Marzetti E., Holloszy J. (2008). Endothelial function after high-sugar-food ingestion improves with endurance exercise performed on the previous day. *The American Journal of Clinical Nutrition*.

[21] Zhu W., Zhong C., Yu Y., Li K. (2007). Acute effects of hyperglycaemia with and without exercise on endothelial function in healthy young men. *European Journal of Applied Physiology*.

[22] Eichner N. Z. M., Gilbertson N. M., Gaitan J. M. (2018). Low cardiorespiratory fitness is associated with higher extracellular vesicle counts in obese adults. *Physiological Reports*.

[23] Solomon T. P. J., Malin S. K., Karstoft K., Kashyap S., Haus J., Kirwan J. P. (2013). Pancreatic *β*-cell Function Is a Stronger Predictor of Changes in Glycemic Control After an Aerobic Exercise Intervention Than Insulin Sensitivity. *The Journal of Clinical Endocrinology & Metabolism*.

[24] Rognmo O., Bjørnstad T. H., Kahrs C. (2008). Endothelial function in highly endurance-trained men: effects of acute exercise. *Journal of Strength and Conditioning Research*.

[25] Birk G. K., Dawson E. A., Batterham A. M. (2013). Effects of exercise intensity on flow mediated dilation in healthy humans. *International Journal of Sports Medicine*.

[26] Tyldum G., Schjerve I., Tjønna A. (2009). Endothelial dysfunction induced by post-prandial lipemia: complete protection afforded by high-intensity aerobic interval exercise. *Journal of the American College of Cardiology*.

[27] Bergholm R., Mäkimattila S., Valkonen M. (1999). Intense physical training decreases circulating antioxidants and endothelium-dependent vasodilatation in vivo. *Atherosclerosis*.

[28] Vincent H. K., Morgan J. W., Vincent K. R. (2004). Obesity exacerbates oxidative stress levels after acute exercise. *Medicine & Science in Sports & Exercise*.

[29] Hu D., Russell R. D., Remash D. (2018). Are the metabolic benefits of resistance training in type 2 diabetes linked to improvements in adipose tissue microvascular blood flow?. *American Journal of Physiology-Endocrinology and Metabolism*.

[30] Barrett E. J., Eggleston E. M., Inyard A. C. (2009). The vascular actions of insulin control its delivery to muscle and regulate the rate-limiting step in skeletal muscle insulin action. *Diabetologia*.

[31] Perkins J. M., Joy N. G., Tate D. B., Davis S. N. (2015). Acute effects of hyperinsulinemia and hyperglycemia on vascular inflammatory biomarkers and endothelial function in overweight and obese humans. *American Journal of Physiology-Endocrinology and Metabolism*.

[32] Dela F., Larsen J. J., Mikines K. J., Ploug T., Petersen L. N., Galbo H. (1995). Insulin-stimulated muscle glucose clearance in patients with NIDDM. Effects of one-legged physical training. *Diabetes*.

[33] Russell R. D., Hu D., Greenaway T. (2017). Skeletal muscle microvascular-linked improvements in glycemic control from resistance training in individuals with type 2 diabetes. *Diabetes Care*.

[34] Malin S. K., Francois M. E., EIchner N. Z. M. (2018). Impact of short-term exercise training intensity on *β*-cell function in older obese adults with prediabetes. *Journal of Applied Physiology*.

[35] Olver T. D., Laughlin M. H., Padilla J. (2019). Exercise and vascular insulin sensitivity in the skeletal muscle and brain. *Exercise and Sport Sciences Reviews*.

[36] Birk G., Dawson E., Atkinson C. (2012). Brachial artery adaptation to lower limb exercise training: role of shear stress. *Journal of Applied Physiology*.

[37] Pyke K. E., Tschakovsky M. E. (2007). Peak vs. total reactive hyperemia: which determines the magnitude of flow-mediated dilation?. *Journal of Applied Physiology*.

[38] McLay K. M., Nederveen J. P., Koval J. J., Paterson D. H., Murias J. M. (2018). Allometric scaling of flow-mediated dilation: is it always helpful?. *Clinical Physiology and Functional Imaging*.

[39] Thijssen D. H. J., Black M. A., Pyke K. E. (2011). Assessment of flow-mediated dilation in humans: a methodological and physiological guideline. *American Journal of Physiology-Heart and Circulatory Physiology*.

[40] Thijssen D. H. J., Bullens L. M., van Bemmel M. M. (2009). Does arterial shear explain the magnitude of flow-mediated dilation?: a comparison between young and older humans. *American Journal of Physiology-Heart and Circulatory Physiology*.

[41] Jahn L. A., Hartline L., RAO N. (2016). Insulin enhances endothelial function throughout the arterial tree in healthy but not metabolic syndrome subjects. *The Journal of Clinical Endocrinology and Metabolism*.

[42] Green D. J., Hopman M. T. E., Padilla J., Laughlin M. H., Thijssen D. H. J. (2017). Vascular adaptation to exercise in humans: role of hemodynamic stimuli. *Physiological Reviews*.

[43] Stoner L., Faulker J., Sabatier M. J. (2013). Is allometry really a panacea for the shortcomings of flow-mediated dilation?. *Journal of Hypertension*.

[44] Nishiyama S. K., Wray D. W., Berkstresser K., Ramaswamy M., Richardson R. S. (2007). Limb-specific differences in flow-mediated dilation: the role of shear rate. *Journal of Applied Physiology*.

[45] Perktold K., Thurner E., Kenner T. (1994). Flow and stress characteristics in rigid walled and compliant carotid artery bifurcation models. *Medical & Biological Engineering & Computing*.

[46] Yeboah J., Crouse J. R., Hsu F., Burke G. L., Herrington D. M. (2007). Brachial flow-mediated dilation predicts incident cardiovascular events in older adults: the Cardiovascular Health Study. *Circulation*.

[47] Moreau K. L., Ozemek C. (2017). Vascular Adaptations to Habitual Exercise in Older Adults: Time for the Sex Talk. *Exercise and Sport Sciences Reviews*.

[48] Shenouda N., Priest S. E., Rizzuto V. I., Mac Donald M. J. (2018). Brachial artery endothelial function is stable across a menstrual and oral contraceptive pill cycle but lower in premenopausal women than in age-matched men. *American Journal of Physiology-Heart and Circulatory Physiology*.

